# The Floral Bottleneck in a Changing Climate: Molecular Mechanisms, Knowledge Gaps, and Future Directions

**DOI:** 10.3390/ijms27072926

**Published:** 2026-03-24

**Authors:** Isabel Marques

**Affiliations:** Forest Research Center (CEF), Associate Laboratory TERRA, School of Agriculture (ISA), University of Lisbon, Tapada da Ajuda, 1349-017 Lisbon, Portugal; isabelmarques@isa.ulisboa.pt

**Keywords:** flowers, floral biology, climate stress, reproductive resilience, abiotic stress, molecular mechanisms, omics, gene editing, epigenetics, pollination

## Abstract

Flowers, the reproductive frontline of plants, are highly sensitive to environmental stresses. Yet, despite their ecological and agricultural importance, the molecular regulation of stress responses in flowers remains comparatively limited. In this context, this review emphasizes how integrative, flower-centered research combining molecular, physiological, and ecological perspectives is essential to safeguard fertility, crop yields, and biodiversity under increasingly variable climates. Advances in single-cell and spatial omics, high-resolution phenotyping, and genome and epigenome editing have the power to unprecedentedly reveal how flowers detect, decode, and respond to environmental stress. By reframing flowers as dynamic molecular decision points rather than passive stress factors, this review outlines a roadmap for flower-centered climate resilience research.

## 1. Introduction

Flowers represent one of the most tightly coordinated developmental transitions in plants, marking the shift from vegetative growth to reproduction, ultimately determining species persistence, genetic diversity, and crop yield [1]. Beyond their primary reproductive function, flowers coordinate a wide array of ecological interactions, shaping the activity of pollinators and other mutualistic partners [2]. Climate-driven changes in temperature and precipitation are already disrupting these interactions, causing phenological mismatches and reduced pollinator visitation, with cascading consequences for ecosystem stability [3,4]. These ecological disruptions stem from the pronounced sensitivity of floral tissues to environmental stress even when vegetative tissues remain comparatively resilient [5,6,7]. For instance, in cereals, short heat waves above 24 °C during anthesis can severely reduce grain through pollen sterility, impaired stigma receptivity, or ovule abortion, while temperatures of 35 °C or higher can result in total crop failure [8,9]. Drought during flowering can also reduce flower lifespan, nectar secretion, and compromise pollination [10,11].

Despite the centrality of flowers to both agricultural and natural systems, the molecular mechanisms that enable flowers to perceive and respond to environmental stresses remain among the least explored in plant biology. Conceptually, the flower has often been regarded as a downstream outcome within the plant life cycle, primarily associated with reproduction, rather than as a central site of environmental perception and adaptive response. In fact, the study of plant stress responses has historically been centered in vegetative tissues. Leaves and roots, which usually grow continuously, are accessibly and metabolically active, became the default models for dissecting stress signaling pathways [12,13]. The flower, by contrast, is transient, developmentally complex, and composed of multiple specialized organs including sepals, petals, stamens, carpels that differ in gene expression, physiology, and stress sensitivity [14,15]. Sampling such ephemeral tissues poses technical challenges for molecular and omics analyses, contributing to a long-standing research bias that has rendered flowers “the poor cousins” of molecular stress biology. But under ongoing climate change, flowers will increasingly face multifactorial stress factors, such as elevated temperatures and atmospheric CO_2_, altered photoperiods and water regimes, which will modify gene-expression networks governing flower responses [16,17,18].

Emerging comparative studies already suggest that flowers and vegetative tissues exhibit distinct physiological responses to environmental stress and may operate under different thresholds. For example, floral tissues of *Dendrobium* display a greater susceptibility to the stress imposed by high light than plant leaves [19]. Likewise, the antioxidant response of *Tagetes erecta* to drought stress showed marked differences between leaves and petals, with a higher enzymatic activity on leaves but a stronger accumulation of non-enzymatic antioxidants on petals [20]. Likewise, under combined water deficit and heat stress, stomata close in soybean leaves but remain open in floral tissues (sepals), contributing to floral cooling and the protection of reproductive organs while also indicating a higher sensitivity of flower organs to stress [21]. Collectively, these physiological divergences also suggest that reproductive tissues may operate under distinct molecular regulatory regimes. In fact, transcriptomic comparisons in soybean plants reveal organ-specific regulation of stress pathways with flowers exhibiting distinct expression patterns, including ABA biosynthesis and degradation genes compared with pods and leaves [22]. These observations suggest that stress-response mechanisms commonly characterized in vegetative tissues may not be directly extrapolated to reproductive organs, particularly given the additional variability among floral organs and developmental stages. Although model systems such as *Arabidopsis thaliana* [23] provide foundational insights, translation of these findings to flowers of major crops and non-model species remains limited, highlighting the need for a flower-centered perspective on climate resilience.

In this context, this review synthesizes the current knowledge on the molecular mechanisms underlying floral stress responses, spanning flowering-time regulation, floral meristem identity, organ-specific stress perception, intracellular signaling, transcriptional and epigenetic reprogramming, and their consequences for reproductive success. By integrating different perspectives, this review identifies key knowledge gaps, evaluates emerging methodological approaches, and outlines future directions for flower-centered research aimed at safeguarding fertility, crop yields, and ecosystem stability.

## 2. Key Molecular Regulators Controlling Flowering Time

Flowering-time regulation represents the earliest developmental stage determining reproductive success [24,25]. Across angiosperms, this molecular regulation relies on a broadly conserved regulatory framework that integrates environmental signals with developmental competence at the shoot apical meristem (SAM), reprogramming meristem identity to initiate floral development (Table 1).

Central among conserved regulatory cues is the florigen pathway, in which the *FLOWERING LOCUS* T (*FT*) functions as a mobile signal produced in leaves under inductive conditions and transported to the SAM, where it promotes floral identity over vegetative growth [26]. In the meristem, *FT* forms a complex with *FLOWERING LOCUS D* (*FD*), activating *APETALA1* (*AP1*) and promoting expression of *SUPPRESSOR OF OVEREXPRESSION OF CO1* (*SOC1*), which subsequently interacts with *AGAMOUS-LIKE 24* (*AGL24*) to induce *LEAFY* (*LFY*) and stabilize floral meristem identity [27,28]. This *FT–SOC1–LFY* signaling axis is also involved in stress responses. For instance, in rice, an *FT-like* gene (*OsFTL10*), is induced by drought and gibberellin signaling, and its overexpression promotes early flowering while enhancing drought tolerance [29]. Thus, hypothetically, manipulation of *FT* genes could improve drought performance. However, without evaluation under field conditions, it remains unclear whether this can be done without yield penalties.

The photoperiodic regulation provides a primary environmental input into this conserved framework, being linked also with stress resilience. Much of our mechanistic understanding of this regulation derives from long-day (LD) model species such as *A. thaliana*, where *CONSTANS* (*CO*) links circadian timing to *FT* activation [30]. Circadian feedback components including *CIRCADIAN CLOCK ASSOCIATED 1* (*CCA1*), *LATE ELONGATED HYPOCOTYL* (*LHY*), and *PSEUDO-RESPONSE REGULATOR* (*PRR*) genes modulate flowering by regulating transcriptional activators such as *GIGANTEA* (*GI*), *SOC1*, and *FT* [31]. This module is a viable target for engineering drought resilience due to its role in the drought-escape response, where it mediates the acceleration of flowering under drought conditions [32]. Nevertheless, while the integration of circadian timing with flowering is broadly conserved, the molecular architecture of photoperiodic control varies substantially among species. LD plants typically employ transcriptional activators such as *CO* to promote flowering, whereas short-day (SD) species rely on repressors such as *Hd1* in rice or *E1* in soybean [33]. Thus, conserved circadian frameworks generate divergent phenological outcomes depending on the ecological strategy, which also suggests divergence in the molecular pathways linked to floral stress resilience.

Vernalization pathways further illustrate the coexistence of conserved functions and molecular diversification. In *Arabidopsis*, prolonged cold induces epigenetic silencing of the floral repressor *FLOWERING LOCUS C* (*FLC*), whose expression is promoted by *FRIGIDA* (*FRI*) [34,35]. In contrast, temperate cereals regulate flowering through *VERNALIZATION* genes (*VRN1*, *VRN2*, and *VRN3*) [36], while beet (*Beta vulgaris*) relies on repression of *BvFT1* alongside activation of *FT*-like genes following cold exposure [37]. Thus, because vernalization pathways control seasonal flowering transitions, tuning regulators such as *FLC*, *FT*, or *VRN* genes may provide opportunities to stabilize reproductive timing under increasingly variable winter climates. However, their complex control and the lineage-specific architecture of these pathways [38] make their manipulation difficult and potentially associated with unintended phenological shifts.

The autonomous pathway also regulates flowering independently of environmental cues through regulators such as *FCA*, *FPA*, *FY*, and *FLD*, which progressively reduce *FLC* activity via RNA processing and chromatin modification [39,40]. Together with vernalization, these regulators ensure that internal age-related signals align with seasonal conditions before floral transition. Changes in these regulators may therefore influence how plants adjust flowering timing under shifting climatic conditions.

Hormonal signaling provides an additional conserved layer integrating growth status with environmental inputs. GAs promote flowering by relieving DELLA-mediated repression of transcriptional activators such as *SOC1* and *LEAFY* at the SAM [41], particularly under marginal photoperiods when canonical cues are weak [42]. Other hormones also contribute to floral development: auxins regulate floral primordium patterning [43], cytokinins enhance meristem competence [44], and brassinosteroids influence flowering timing partly through modulation of *FLC* expression [45]. Manipulation of these hormones could therefore improve floral resilience. However, the strong interconnectedness of hormonal pathways suggests that manipulating these regulators to enhance floral resilience may also involve trade-offs with growth and developmental timing.

Flowering-time regulation is further refined by post-transcriptional, epigenetic, and metabolic mechanisms that provide regulatory plasticity. Non-coding RNAs add an important regulatory layer linking developmental timing with environmental responsiveness. MicroRNAs such as miR156 and miR159 regulate SPL and MYB transcription factors, coordinating flowering competence with plant age and stress-responsive signaling networks [46,47]. In particular, the conserved miR156–SPL module integrates developmental transitions with abiotic stress responses, suggesting that modulation of flowering timing may contribute to stress avoidance. Long non-coding RNAs, including COOLAIR, COLDAIR, and COLDWRAP, regulate epigenetic silencing of *FLC* during vernalization, linking environmental history with reproductive timing [48]. Chromatin-based regulation through Polycomb-mediated H3K27me3 and Trithorax-mediated H3K4me3 stabilizes transcriptional states at key regulators such as *FLC*, *FT*, and *LFY* [49,50], enabling flowering decisions to persist after environmental cues and potentially contributing to stress memory. Chromatin remodeling complexes such as SWR1-C further integrate temperature sensing via regulation of the H2A.Z histone variant [51]. In parallel, metabolic signals such as trehalose-6-phosphate (T6P) communicate carbohydrate availability to the meristem by activating *FT* and *SPL* transcription factors [52], linking plant energy status with reproductive timing under stress conditions that constrain carbon supply.

Altogether, these pathways reveal a central feature of flowering-time regulation: deeply conserved developmental modules that operate within highly variable regulatory architectures. This variability allows for the evolutionary tuning of floral responses to local climates but also complicates predictions of reproductive responses under global change. For instance, despite extensive molecular characterization, it remains debated whether *FT* regulators influence stress resilience directly through interactions with stress signaling networks or indirectly by shifting developmental timing relative to environmental stress exposure. From a breeding perspective, this dual role suggests that manipulating *FT* regulators may enhance resilience either by aligning reproductive stages with favorable environmental windows or by exploiting regulatory crosstalk between developmental and stress-response pathways [53]. Targeted tuning of conserved modules, rather than introduction of entirely new stress traits, may therefore represent a promising strategy for improving crop performance under climate variability.

## 3. Floral Meristem Identity and Organ Specification

Once the floral transition is achieved, and in response to *CO* expression, the shoot apical meristem activates floral meristem identity genes, most prominently *LEAFY* (*LFY*) and *APETALA* (*AP1*), which reprogram the meristem from a vegetative to a floral fate [54,55]. The subsequent spatial and temporal patterning of floral organs is governed by the ABCDE model of MADS-box gene interactions. In this framework, Class A genes (*AP1*, *AP2*) specify sepals and, together with Class B genes (*APETALA3*: *AP3*, *PISTILLATA*: *PI*), specify petals [56,57]. Class B in combination with Class C (*AGAMOUS*, *AG*) defines stamens, whereas Class C alone determines carpel identity. Class D genes, such as *SEPALSTICK* (*STK*) and *SHATTERPROOF* (*SHP*), control ovule development, while Class E genes (*SEP1*–4) act as essential co-factors that enable the formation of higher-order transcriptional complexes [58]. Originally characterized in *Arabidopsis* and *Antirrhinum*, the ABCDE regulatory framework is broadly conserved across angiosperms, although gene duplication and subsequent subfunctionalization have diversified these floral regulatory networks. For example, in orchids, the duplication of B-class genes (*AP3*/*PI* homologs) followed by subfunctionalization produced the “orchid code,” possibly enabling the formation of the highly specialized labellum [59]. In grasses, the ABCDE program is redeployed in a modified form and instead of petals and sepals, orthologs of A- and B-class genes specify lemma, palea, and lodicules [60].

Because MADS-box genes generate highly specialized and metabolically active reproductive tissues, stress disproportionately affects organs specified by these networks. Such developmental instability is illustrated, for example, by pistillody, the transformation of stamens into pistil-like structures, primarily associated with altered activity of Class B genes controlling male organ identity and fertility [61]. In *Arabidopsis*, drought-induced early flowering involves Class A genes, including *AP1*, *SOC1*, *FLC*, and *SVP*, indicating that stress conditions can reshape developmental programs governing floral identity [62]. In tomato, salt stress enhances the expression of *SFT* (*SINGLE-FLOWER TRUSS-AP1*) genes once plants attain flowering competence, reducing biomass but accelerating the transition to flowering [63].

Importantly, ABCDE genes do not appear to function as canonical stress-response regulators; rather, they establish the organ-specific developmental contexts within which stress perception, signaling, and damage occur. Consequently, variation in floral developmental programs may influence reproductive resilience indirectly by determining which tissues are most exposed or sensitive during critical developmental windows. However, several fundamental questions remain unresolved. First, it is still unclear whether abiotic stress directly perturbs organ identity networks or whether observed floral abnormalities arise primarily from downstream physiological failure. Second, most mechanistic insights derive from model species, leaving the extent of conservation and functional divergence across most crops and non-model angiosperms largely unknown. Third, flower tissues are highly heterogeneous and developmentally transient, making it difficult to isolate specific organs or cell types at comparable developmental stages during stress exposure. Critical reproductive cell types such as meiocytes, tapetal cells, or ovule tissues remain technically challenging to access, limiting mechanistic insight into tissue-specific vulnerability [64]. Addressing these gaps will require integrating developmental genetics with tissue-resolved stress biology, representing a major frontier for understanding floral vulnerability under climate change.

## 4. Stress Perception and Signaling in Floral Tissues

One of the major challenges in floral research is that stress perception in flowers is both spatially and temporally heterogeneous because the different floral tissues have distinct sensory capacities and damage thresholds that shift across developmental stages [65,66]. These spatial differences become evident in the contrasting vulnerability of floral organs. Ovules and stigmatic tissues are usually considered to be more resilient than male organs, although recent evidence indicates that their receptivity might also decline even under mild desiccation [67,68]. Temporal heterogeneity further arises from strong stage dependence in stress sensitivity with meiosis and early microspore development being among the most heat-labile phases, whereas fertilization and early embryogenesis are particularly susceptible to cold and water stress [69]. Together, these “critical windows” delineate the developmental periods during which environmental stress most strongly determine floral success or failure. Thus, stress perception in flowers is inseparable from organ identity and developmental timing, rather than being uniformly distributed across the floral structure, helping explain why reproductive tissues frequently fail even when vegetative organs remain functional.

Among the abiotic factors influencing these sensitivities, temperature stands out as one of the most consequential cues for floral performance. Flowers perceive thermal variation both through rapid biophysical changes in membrane fluidity, which alter ion fluxes and signal transduction, and through temperature-sensitive photoreceptors, particularly PHYB, whose thermal reversion interferes with *CONSTANS–FT* signaling and thus with the timing of reproductive development [70]. Floral tissues also detect stress through cell wall integrity receptors, including FERONIA (FER) and THESEUS1 (THE1) [71], which sense changes in wall tension, pectin crosslinking, and turgor pressure before irreversible cellular damage occurs [72]. In anthers, mechanoperception contributes to the timing and robustness of dehiscence and interacts with hydration status [73]. Together with ionic and osmotic sensors, these mechanisms enable local stress detection at the organ and cellular scale, suggesting that flowers possess partially autonomous sensing systems that may prioritize reproductive protection independently of whole-plant conserved signaling.

**Figure 1:**
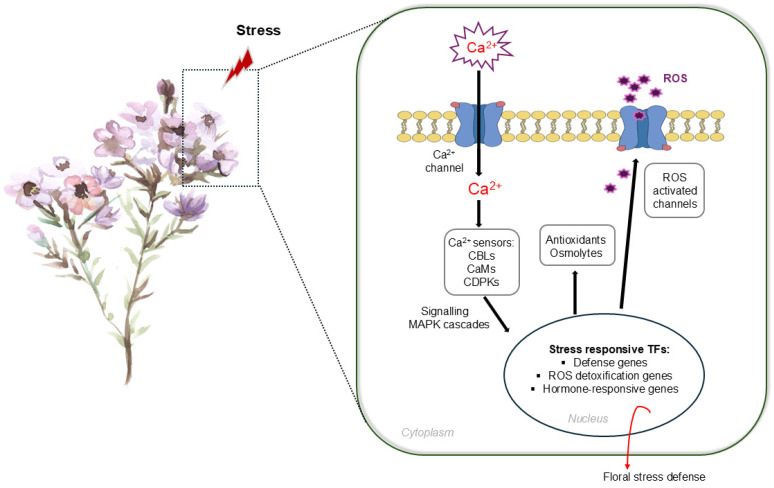
Simplified model of Ca^2+^–ROS–MAPK signaling in floral stress responses. Stress perception induces Ca^2+^ influx and ROS production via plasma membrane channels and RBOHs. Ca^2+^ sensors (CaMs, CBLs, CDPKs) and MAPK cascades relay signals to the nucleus, activating stress-responsive transcriptional programs, including defense, redox homeostasis, and hormone-regulated programs, ultimately promoting floral stress tolerance.

Once perceived, stress is transduced into intracellular signals primarily through a conserved Ca^2+^–ROS–MAPK signaling module shared across plant tissues (Figure 1). Transient Ca^2+^ spikes decoded by calmodulins (CaMs), calcineurin B-like proteins (CBLs) and Ca^2+^-dependent protein kinases (CDPKs) interact with ROS produced by NADPH oxidases (RBOHs) to amplify signaling responses [74]. These messengers activate mitogen-activated protein kinase (MAPK) cascades such as MPK3/6 and their upstream kinases MKK4/5, which in turn modulate transcription factor activity and initiate protective gene expression programs. Although this signaling architecture is evolutionarily conserved, its developmental interpretation appears to differ in floral tissues, where activation frequently results in developmental arrest or reduced fertility rather than in the acclimatory growth responses observed in vegetative organs [75].

Beyond the cytosol, organelles act as semi-autonomous stress sensors that directly influence floral reproductive resilience. Mitochondrial AOX-mediated buffering mitigates ROS accumulation and supports energy supply during stress, processes essential for pollen viability and fertilization [76]. Chloroplast-derived retrograde signals adjust nuclear transcription to rebalance metabolism under fluctuating environments [77], whereas ER activation of the unfolded protein response (UPR) via bZIP17 and bZIP60 preserves proteostasis in highly secretory reproductive tissues [78]. Failure of these organelle-based buffering systems is therefore increasingly associated with pollen sterility, impaired fertilization, and floral abortion under climate stress.

Hormonal signals act downstream of early stress perception pathways as rapid modulators that rebalance growth and protection in floral tissues, functioning differently from their classical developmental roles in flowering-time regulation. Abscisic acid (ABA) accumulates rapidly under drought or heat stress, promoting cellular protection mechanisms and osmotic adjustment, but often at the cost of reduced reproductive growth [79]. Jasmonates (JAs) activate defense-like responses in anthers and pistils but excessive signaling can accelerate tapetal degeneration or induce floral organ abortion under severe stress, illustrating a narrow balance between protection and reproductive loss [80]. Ethylene regulates stigma senescence and pollen–pistil interactions, frequently accelerating reproductive shutdown when stress becomes prolonged [81]. Conversely, suppression of gibberellin (GA) biosynthesis stabilizes DELLA proteins, temporarily restraining meristematic activity and delaying developmental progression until favorable conditions return [82].

All these sensing and signaling networks form a conserved molecular framework for stress detection, yet their developmental interpretation in floral tissues remains incompletely understood. Therefore, a major unresolved question concerns how local organ-level stress perception is integrated with systemic plant signaling. Furthermore, the transient and highly heterogeneous nature of floral tissues poses significant technical challenges for experimental investigation, including difficulties in stage-specific sampling, limited cell-type resolution, and underrepresentation of non-model species in omics datasets. These limitations contribute to ongoing debates about whether floral resilience primarily depends on enhanced stress tolerance mechanisms or on developmental buffering strategies that shift or shorten vulnerable reproductive windows. Future research integrating spatially resolved omics, live imaging, and comparative studies across diverse species will be essential to determine how conserved signaling modules are rewired in reproductive contexts and how these processes can be leveraged to stabilize fertility under climate change.

## 5. Molecular Reprogramming of Flowers Under Climate Stress: How Much and for How Long?

Once early stress signals have been perceived and transduced, floral tissues transition into a deeper layer of molecular reprogramming that determines which genes are activated or repressed and how persistently these changes are maintained. This process forms a continuum ranging from transient responses to transgenerational memory. Three broad categories of stress-memory genes have been proposed: (i) transcriptional-memory genes, which maintain altered expression after recovery; (ii) epigenetic-memory genes, in which stress-induced chromatin modifications outlast the stimulus even in the absence of sustained transcriptional changes; and (iii) delayed-memory genes, which retain stress information and activate only later responses. Importantly, these memory layers do not correspond to specific floral organs or developmental stages but instead operate as temporal regulatory mechanisms acting across multiple tissues.

At the transcriptional level, stress induces a rapid reprioritization of gene expression favoring flower protection over growth [83]. During heat stress in rice, young anthers strongly upregulate Heat Shock Factors (HSFs) within minutes, illustrating the fast and reversible nature of first-wave transcriptional reprogramming [84]. Rapid transcriptional responses have also been recorded in *Brassica napus* anthers exposed to a high temperature of 40 °C for 5, 15, and 30 min [85]. These early responses operate on short timescales and typically dissipate once stress subsides, unless they are stabilized by downstream chromatin-level regulation.

Epigenetic remodeling provides a slower and potentially more persistent layer of regulation [86]. Comparable chromatin modifications have been observed under heat and drought stress in reproductive tissues, where histone changes in developing cereal anthers modulate later fertility outcomes [87], and seasonal epigenetic signatures may persist in floral meristems across years [88]. Because floral meristems give rise to gametophytic tissues, some stress-associated epigenetic states established during flowering can influence reproductive performance beyond the initial stress episode [89]. However, a major controversy in the field concerns whether stress-induced epigenetic changes represent adaptive memory mechanisms or non-adaptive consequences of developmental disruption.

Proteostasis constitutes an additional buffering mechanism, acting on shorter timescales. In rice, the tapetum-specific chaperone HSP17.4-CI protects developing pollen by preventing protein aggregation under thermal stress [90]. Unlike epigenetic regulation, these proteostatic responses operate over minutes to hours and are largely reversible once protein-folding capacity is restored, providing rapid but transient protection that complements longer-term transcriptional and chromatin adjustments.

Metabolic reconfiguration further modulates stress persistence by linking cellular energy status to developmental progression. For instance, in some species, drought triggers the activation of the SnRK1 energy sensor suppressing growth-related transcription and delaying reproductive metabolism until carbohydrate availability is restored [91]. These metabolic switches typically persist longer than transcriptional bursts but reset once balance is recovered.

A final and longer-term layer of stress-induced reprogramming may extend beyond the individual plant, affecting subsequent generations. In *Brassica napus*, drought during flowering can induce methylation changes in ovule and gametophyte tissues that are transmitted to the next generation, priming progeny for altered stress sensitivity [92]. Such transgenerational effects appear particularly relevant in perennials and polycarpic species, where reproductive history feeds back into future flowering cycles.

Yet, despite growing evidence that flowers undergo multilayered molecular reprogramming under stress, the adaptive significance and stability of these responses remain incompletely understood. Another limitation lies in the strong reliance on controlled laboratory studies, which rarely capture the combined and fluctuating stresses experienced during flowering in natural or agricultural systems. Under field conditions, repeated stress cycles may overwrite or reset molecular memory, challenging the assumption that stress priming consistently enhances resilience [93]. Moreover, stress memory may involve trade-offs: prolonged activation of protective pathways can delay development or reduce reproductive allocation, potentially lowering yield despite increased stress tolerance [94]. From a breeding perspective, these uncertainties limit the use of stress memory as a selectable trait, as epigenetic states may not remain stable across environments or generations, complicating their integration into crop improvement programs. Future research must therefore determine under which ecological and developmental contexts floral stress memory promotes adaptive resilience rather than reproductive constraint, requiring multigenerational experiments, field validation, and integration of spatially resolved omics approaches. Ultimately, the key challenge is not identifying additional memory mechanisms but understanding when and where they meaningfully enhance reproductive success under climate variability.

## 6. Consequences of Floral Stress for Reproductive Success, Yield, and Ecosystem Stability

The severity of the outcomes of floral stress depends not only on the intensity of the disturbance, but also on how long the molecular reprogramming described in the previous section persists. When buffering systems are overwhelmed or stress endures long enough to prevent molecular reset, transient protective responses give way to structural and developmental injuries [95,96]. The result is a progressive cascade from cellular dysfunction to failure, and ultimately to a decline in reproductive success.

**Figure 2:**
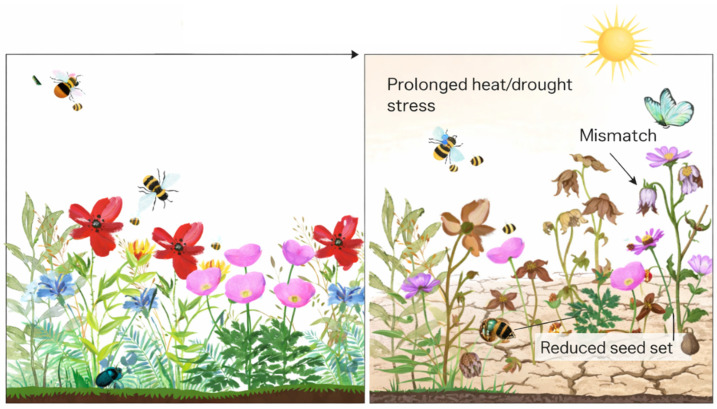
Scaling consequences of floral stress from reproductive failure to ecosystem instability. (**Left**): Under favorable environmental conditions, floral tissues maintain cellular and physiological homeostasis, allowing for coordinated flowering, effective pollinator interactions, and successful fertilization, resulting in stable reproductive output. (**Right**): Under prolonged stress such as heat or drought, buffering capacity is exceeded, leading to floral dysfunction, including flower wilting, pollen and stigma impairment, and increased flower and seed abortion. Temporal mismatches between flowering and pollinator activity further exacerbate reproductive failure.

At the plant scale, such organ-level failures translate directly into reduced fruit or seed set, even when vegetative tissues remain apparently unaffected [97,98]. In major cereal crops such as wheat, even a single day of high temperatures (35 °C) at anthesis can reduce grain number and yield by up to 33% and 35%, respectively [99]. In sorghum, mean daily temperatures above 25 °C sustained for ten days can dramatically reduce fertility, reaching complete sterility at 37 °C when heat stress occurs at the onset of panicle emergence [100]. These risks are expected to increase under future climate scenarios, as phenological projections suggest a growing likelihood of mismatches or failures during flowering, although responses vary considerably among species depending on their adaptive capacity [101]. The consequences of reproductive stress extend beyond agricultural systems. For example, drought stress negatively affects plant growth, floral bud formation, petal size, nectar production, and pollinator visitation in the monoecious *Cucurbita pepo*, with impacts being more severe on female than on male flowers, as most female flowers abort before anthesis [102]. At the end, recurrent reproductive failure reduces population recruitment and genetic turnover [103] and exacerbates mismatches with pollinator activity, particularly in species with narrow reproductive windows [104]. These cascading effects illustrate how molecular perturbations initiated within floral tissues can scale up to influence population dynamics and ecosystem stability (Figure 2).

Despite growing recognition of these multiscale consequences, several key uncertainties remain. A central unresolved question is whether moderate reproductive injury is fully reversible or whether cumulative sublethal damage across flowering cycles contributes to long-term fertility decline. Additionally, the extent to which combined stresses (e.g., heat and drought) shift thresholds for irreversible damage relative to single stress exposure remains poorly quantified, especially because the temperature, duration, and frequency required to cause permanent sterility varies widely among species/genotype. This variability also complicates prediction of reproductive resilience under fluctuating climate regimes. Integrating molecular markers of stress perception with demographic and yield-based measurements represents a critical research priority. Thus, future work must combine high-resolution developmental analyses, genotype comparisons, and multiyear field validation to determine when floral stress responses promote adaptive resilience and when they signal the onset of irreversible reproductive loss.

## 7. Potential Biotechnological Interventions Targeting Floral Stress Resilience

The molecular vulnerabilities described previously suggest that climate-resilience strategies should move beyond general whole-plant stress tolerance toward the targeted protection of reproductive tissues. One promising route operates through transcriptional reprogramming. Stabilizing key transcriptional hubs may prevent the collapse of protective gene networks during stress [105]. Marker-assisted breeding and QTL introgression have identified regulators that maintain tapetal homeostasis, ROS buffering, and carbohydrate delivery to reproductive tissues [106]. For example, heat-tolerance QTLs in rice sustain HSP, antioxidants, and sugar-transporter expression, improving pollen viability under elevated temperatures [107]. Similarly, drought-responsive *NAC* and *DREB* alleles in wheat help preserve ovule function under water deficit [108]. Yet, these regulatory strategies face important limitations. Many stress-responsive transcription factors exhibit pleiotropic effects, influencing vegetative growth, phenology, or resource allocation [109]. However, QTL effects identified under controlled environments may weaken under field heterogeneity, and transcriptional buffering does not necessarily prevent fertility collapse when stress exceeds physiological thresholds. Thus, while upstream regulatory stabilization is conceptually attractive, its durability under extreme or repeated climate events remains uncertain.

A second strategy builds on chromatin-based “priming” of reproductive tissues. Stress-induced histone modifications may facilitate faster reactivation of defense genes upon re-exposure. For instance, mild heat during *Arabidopsis* anther development increases H3K4me3 accumulation at HSP loci, enhancing subsequent pollen resilience [110]. Such epigenetic priming suggests potential for plant engineering. However, the stability and heritability of induced chromatin states are variable, limiting their immediate utility in breeding programs, where trait stability across environments is essential.

Metabolic engineering offers a third avenue by enhancing carbon allocation and osmoprotectant accumulation to sustain gametophyte viability [111,112]. Manipulating sucrose transporters, invertases, or compatible solute pathways may buffer reproductive tissues during transient stress. Nevertheless, altering metabolic fluxes risks trade-offs with vegetative growth or grain filling, and source–sink relationships are highly context-dependent. Field validation of such strategies, particularly under combined stress scenarios, remains limited.

Hormonal modulation provides additional opportunities, but it also introduces further complexity. Fine-tuning ABA, GA, or jasmonate pathways in reproductive tissues may reduce stress-induced abortion or enhance fertilization efficiency [113,114]. However, hormonal networks are deeply interconnected, and modifying one node may disrupt developmental timing or reproductive synchrony. Spatial precision is therefore critical. The use of tissue-specific promoters (e.g., LAT52 for pollen or DD45 for ovules) enables targeted protection while minimizing pleiotropic penalties [109,115,116].

Importantly, biotechnological strategies should complement rather than replace natural genetic variation. Landraces and wild relatives often harbor alleles conferring partial reproductive resilience that can be introgressed through conventional or genomic-assisted breeding. Integrating natural diversity with gene editing, promoter engineering, or marker-assisted selection may provide more stable and socially acceptable solutions than transgenic approaches alone. Several case studies already demonstrate measurable reproductive gains: introgression of heat-tolerance QTLs in rice improves spikelet fertility [117], drought-responsive alleles in chickpea sustain ovule viability and seed set [118], and CRISPR-mediated editing of ABA receptors in tomato flowers reduces drought-induced ovule abortion [119].

**Figure 3:**
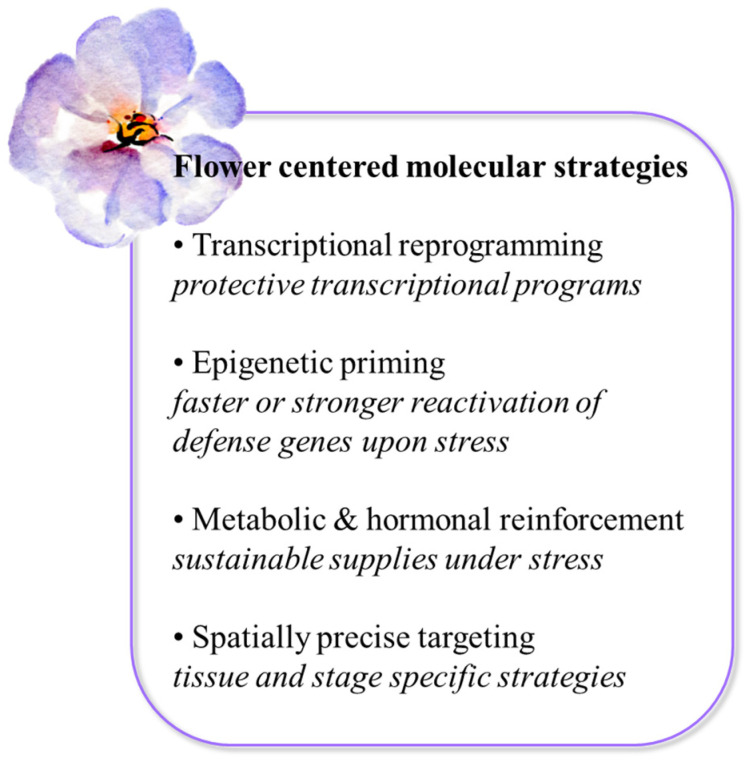
Conceptual representation of flower-centered main molecular interventions designed to preserve reproductive function under stress.

Altogether, enhancing floral resilience will likely require integrated strategies combining natural genetic diversity, precise molecular interventions, and conventional breeding frameworks (Figure 3). A major open question is whether tissue-specific engineering can consistently buffer reproductive tissues without incurring hidden yield penalties under multi-stress conditions. Bridging molecular innovation with multiyear field validation and socio-economic feasibility will therefore determine whether flower-centered resilience strategies translate into climate-ready crops.

## 8. Emerging Tools and Future Directions for Flower-Focused Climate Resilience Research

Because flowers integrate numerous environmental and endogenous cues within short temporal windows, protecting them requires a new generation of tools capable of resolving stress responses at cellular precision and acting within those windows.

First, finer molecular resolution through single-cell and spatial omics now uncovers the exact transcriptional, epigenetic, and metabolic states of individual floral cell types [120]. In *Arabidopsis*, single-cell spatial transcriptomics of floral tissues were able to identify epidermal markers (*AT2G37540*), vascular-expressed genes (*FT*; *AT1G65480*) and carpel-expressed genes *AGAMOUS-like 8/FRUITFULL* [121]. These approaches can move floral stress research beyond organ-level averages, enabling direct identification of the specific cells and developmental moments at which resilience is gained or lost.

High-resolution phenotyping complements molecular insights with functional readouts. Thermal and hyperspectral imaging, fluorescent ROS and metabolite probes, and automated assays for pollen viability, ovule integrity, nectar volume, and volatile emission now permit quantitative monitoring of floral function in real time [122]. Such phenotyping frameworks are essential for linking molecular responses to reproductive performance rather than surrogate stress markers. However, floral traits are ephemeral and highly stage-specific, making standardized capture under fluctuating field conditions challenging. Additionally, most phenotyping platforms have been validated under single-stress regimes, whereas flowering increasingly occurs under combined heat, drought, and atmospheric variability. Developing scalable phenotyping systems that accurately quantify reproductive thresholds under multifactorial stress remains a significant bottleneck.

A third frontier lies in molecular precision and cell-type–specific gene control. Innovation has focused on where resilience should be targeted. Yet, the most vulnerable phases of floral development are often brief and irreversible, suggesting that constitutive stress tolerance may be less effective than temporally restricted buffering during critical windows. Thus, the next generation of interventions should focus on when protective mechanisms must be engaged. This shift from static tolerance to time-gated protection redefines how floral resilience may be engineered. Nevertheless, implementing temporally programmable circuits in crops presents technical challenges, including promoter specificity, off-target effects, and regulatory approval constraints.

Looking further ahead, integrative modeling and ecological coupling may close the loop between molecular processes and ecosystem function. Artificial intelligence frameworks are beginning to merge omics, phenotyping, and environmental datasets to forecast floral performance [122]. However, predictive models remain limited by biased training datasets derived largely from controlled environments and model species. Integrating longitudinal field data, multi-stress scenarios, and demographic outcomes into modeling frameworks is an unresolved computational and conceptual challenge.

Ultimately, emerging technologies will reshape research strategies only if they are integrated into coordinated, flower-centered pipelines that combine cellular-resolution diagnostics, multiscale phenotyping, predictive modeling, and ecological validation. Key priorities include: (i) defining cellular and developmental thresholds for irreversible reproductive damage; (ii) extending genomic and spatial resources to crop and non-model species; and (iii) integrating molecular innovation with breeding and field validation. Without such integration, increased resolution alone will not translate into improved reproductive resilience under climate variability.

## 9. Conclusions

Despite their central role in reproduction and ecosystem function, floral stress responses have historically remained a marginal topic in plant molecular biology. Advances in multi-omics and precision phenotyping revealed that floral resilience is not governed by single genes, but by dynamic regulatory networks that operate with strict spatial and temporal specificity. These discoveries reframe flowers not as passive stress casualties, but as highly regulated developmental decision points that determine whether reproduction succeeds under climate pressure.

This review highlights how flowering time regulation, floral meristem identity, organ-specific stress perception, intracellular signaling, and molecular reprogramming determine whether reproductive development proceeds, pauses, or fails under environmental stress. Importantly, stress signaling emerges not as discrete floral stages, but as cross-cutting regulatory layers whose persistence in time, rather than localization in space, dictates reproductive outcomes. Yet substantial uncertainties remain regarding thresholds of irreversibility, multi-stress integration, and the stability of stress-induced molecular states across environments and generations.

The challenge is no longer to catalog additional stress-responsive genes, but to translate mechanistic understanding into predictive and protective strategies. This translation must integrate molecular innovation with natural genetic variation, genomic-assisted breeding, and field validation to ensure that resilience traits remain stable under realistic climatic complexity. This requires a shift from constitutive stress tolerance toward spatially and temporally precise interventions that safeguard reproductive tissues during their most vulnerable developmental phases. As climate extremes intensify, the molecular logic of the flower will increasingly define the boundary between reproductive collapse and adaptation. Meeting this challenge will require coordinated efforts across molecular biology, breeding, ecology, and computational modeling, supported by multiscale field validation. By prioritizing flowers as a focal point for integrative research, it becomes possible not only to understand plant reproductive failure, but to anticipate and mitigate it in an increasingly unstable world.

## Figures and Tables

**Table 1:** Major spatial, developmental, and molecular levels at which flowers perceive, integrate, and respond to environmental stress. For each floral level, key tissues, dominant stressors, representative molecular regulators, characteristic response timescales, and functional outcomes are indicated. Timescales reflect the approximate persistence of stress responses, ranging from rapid signaling events to long-term epigenetic memory. Molecular regulators are illustrative examples mainly focused on annual species and do not represent exhaustive inventories.

Floral Level	Key Tissues	Dominant Stressors	Molecular Regulators	Timescale	Outcome
Floral induction	SAM	Temperature fluctuations, photoperiod shifts	*FT*, *SOC1*, *LFY*, *FLC*	Days–weeks	Timing of flowering
Floral identity	Meristem	Temperature variability, developmental perturbation	ABCDE MADS-box genes	Developmental	Organ specification
Male gametogenesis	Anther, pollen	Heat, drought, oxidative stress	HSPs, tapetal TFs	Hours–days	Pollen viability
Female gametogenesis	Ovule, stigma	Drought, heat	Cell-wall TFs, hormones	Hours–days	Fertilization success
Anthesis/Reproduction	Whole flower	Heat, desiccation	Hormones, volatiles	Hours	Pollination efficiency

## Data Availability

No new data were created or analyzed in this study. Data sharing is not applicable to this article.

## References

[1] Mei Z., de Groot G.A., Kleijn D., Dimmers W., van Gils S., Lammertsma D., van Kats R., Scheper J. (2021). Flower availability drives effects of wildflower strips on ground-dwelling natural enemies and crop yield. Agric. Ecosyst. Environ..

[2] Zu K., Wang Z., Chen F., Lenoir J., Fang X., Wang F., Bu W., Li J., Luo Y., Wang Y. (2025). Climate-Driven Variability in Flowering Phenology Changes Across Subtropical Mountains: Traits, Elevation Shifts, and Biogeographic Patterns. Glob. Change Biol..

[3] Peng S., Ellison A.M., Davis C.C. (2025). Climate change intensifies plant–pollinator mismatch and increases secondary extinction risk for plants in northern latitudes. Proc. Natl. Acad. Sci. USA.

[4] Chachar S., Ahmed N., Hu X. (2025). Future-proofing ornamental plants: Cutting-edge strategies for drought resistance and sustainability. Physiol. Plant..

[5] Benitez-Alfonso Y., Soanes B.K., Zimba S., Sinanaj B., German L., Sharma V., Bohra A., Kolesnikova A., Dunn J.A., Martin A.C. (2023). Enhancing climate change resilience in agricultural crops. Curr. Biol..

[6] Giordano M., Petropoulos S.A., Rouphael Y. (2021). Response and Defence Mechanisms of Vegetable Crops against Drought, Heat and Salinity Stress. Agriculture.

[7] Khan N.A., Owens L., Nuñez M.A., Khan A.L. (2025). Complexity of combined abiotic stresses to crop plants. Plant Stress.

[8] Zhu T., Cheng X., Li C., Li Y., Pan C., Lu G. (2025). Decoding plant thermosensors: Mechanism of temperature perception and stress adaption. Front. Plant Sci..

[9] El-Sappah A.H., Rather S.A., Wani S.H., Elrys A.S., Bilal M., Huang Q., Dar Z.A., Elashtokhy M.M.A., Soaud N., Koul M. (2022). Heat Stress-Mediated Constraints in Maize (*Zea mays*) Production: Challenges and Solutions. Front. Plant Sci..

[10] Phillips B.B., Shaw R.F., Holland M.J., Fry E.L., Bardgett R.D., Bullock J.M., Osborne J.L. (2018). Drought reduces floral resources for pollinators. Glob. Change Biol..

[11] Defalque C., Laeremans J., Drugmand J., Tcheutchoua C.F., Meng Y., Zhou M., Zhang K., Quinet M. (2025). Drought and High Temperatures Impact the Plant–Pollinator Interactions in *Fagopyrum esculentum*. Plants.

[12] Carter G.A. (1994). Ratios of leaf reflectances in narrow wavebands as indicators of plant stress. Remote Sens..

[13] Kalra A., Goel S., Elias A.A. (2024). Understanding role of roots in plant response to drought: Way forward to climate-resilient crops. Plant Genome.

[14] Wu Q., Wu Y., Li R., Cao H., Li Z., Li Q., Zhou L. (2025). Research Progress on the Regulation of Plant Floral Organ Development by the MADS-box Gene Family. Int. J. Mol. Sci..

[15] Mohanty J.N., Sahoo S., Mishra P. (2022). A genetic approach to comprehend the complex and dynamic event of floral development: A review. Genom. Inform..

[16] Springer C.J., Orozco R.A., Kelly J.K., Ward J.K. (2008). Elevated CO_2_ influences the expression of floral-initiation genes in *Arabidopsis thaliana*. New Phytol..

[17] Li Z., Wang S., Wang Y., Zhang H., Liu L., Su S., Lin S. (2025). The effects of biotic stress on the sexual reproduction process of flowering plants. PeerJ.

[18] Osnato M., Cota I., Nebhnani P., Cereijo U., Pelaz S. (2022). Photoperiod Control of Plant Growth: Flowering Time Genes Beyond Flowering. Front. Plant Sci..

[19] He J., Khoo G.H., Hew C.S. (1998). Susceptibility of CAM *Dendrobium* leaves and flowers to high light and high temperature under natural tropical conditions. Environ. Exp. Bot..

[20] Tian Z., Wang F., Zhang W., Liu C., Zhao X. (2012). Antioxidant mechanism and lipid peroxidation patterns in leaves and petals of marigold in response to drought stress. Hortic. Environ. Biotechnol..

[21] Sinha R., Zandalinas S.I., Fichman Y., Sen S., Zeng S., Gómez-Cadenas A., Joshi T., Fritschi F.B., Mittler R. (2022). Differential regulation of flower transpiration during abiotic stress in annual plants. New Phytol..

[22] Sinha R., Shostak B., Induri S.P., Sen S., Zandalinas S.I., Joshi T., Fritschi F.B., Mittler R. (2023). Differential transpiration between pods and leaves during stress combination in soybean. Plant Physiol..

[23] Su Z., Ma X., Guo H., Sukiran N.L., Guo B., Assmann S.M., Ma H. (2013). Flower Development under Drought Stress: Morphological and Transcriptomic Analyses Reveal Acute Responses and Long-Term Acclimation in *Arabidopsis*. Plant Cell.

[24] Baral R., Vainer A., Melzer S., Hause B., Panda S. (2025). “Bud to bloom”—Hormonal coordination in floral initiation. Plant Biol..

[25] Yang M., Lin W., Xu Y., Xie B., Yu B., Chen L., Huang W. (2024). Flowering-time regulation by the circadian clock: From Arabidopsis to crops. Crop J..

[26] Turck F., Fornara F., Coupland G. (2008). Regulation and identity of florigen: Flowering Locus T moves center stage. Annu. Rev. Plant Biol..

[27] Yoo S.K., Chung K.S., Kim J., Lee J.H., Hong S.M., Yoo S.J., Yoo S.Y., Lee J.S., Ahn J.H. (2005). *CONSTANS* Activates *SUPPRESSOR OF OVEREXPRESSION OF CONSTANS 1* through *FLOWERING LOCUS T* to Promote Flowering in Arabidopsis. Plant Physiol..

[28] Liu C., Chen H., Er H.L., Soo H.M., Kumar P.P., Han J.H., Liou Y.C., Yu H. (2008). Direct interaction of *AGL24* and *SOC1* integrates flowering signals in *Arabidopsis*. Development.

[29] Fang M., Zhou Z., Zhou X., Yang H., Li M., Li H. (2019). Overexpression of *OsFTL10* induces early flowering and improves drought tolerance in *Oryza sativa* L.. PeerJ.

[30] Suárez-López P., Wheatley K., Robson F., Onouchi H., Valverde F., Coupland G. (2001). CONSTANS mediates between the circadian clock and the control of flowering in *Arabidopsis*. Nature.

[31] Lu S.X., Webb C.J., Knowles S.M., Kim S.H.J., Wang Z., Tobin E.M. (2012). *CCA1* and *ELF3* interact in the control of hypocotyl length and flowering time in *Arabidopsis*. Plant Physiol..

[32] Brandoli C., Petri C., Egea-Cortines M., Weiss J. (2020). Gigantea: Uncovering New Functions in Flower Development. Genes.

[33] Lu S., Dong L., Fang C., Liu S., Kong L., Cheng Q., Chen L., Su T., Nan H., Zhang D. (2020). Stepwise selection on homeologous PRR genes controlling flowering and maturity during soybean domestication. Nat. Genet..

[34] Shindo C., Lister C., Crevillen P., Nordborg M., Dean C. (2006). Variation in the epigenetic silencing of FLC contributesto natural variation in *Arabidopsis* vernalization response. Genes Dev..

[35] Montez M., Zhu D., Huertas J., Maristany M.J., Rutjens B., Nielsen M., Collepardo-Guevara R., Dean C. (2025). Cold-induced nucleosome dynamics linked to silencing of *Arabidopsis* FLC. Nat. Commun..

[36] Kennedy A., Geuten K. (2020). The Role of FLOWERING LOCUS C Relatives in Cereals. Front. Plant Sci..

[37] Kroupin P.Y., Kroupina A.Y., Karlov G.I., Divashuk M.G. (2023). Root Causes of Flowering: Two Sides of Bolting in Sugar Beet. Agronomy.

[38] Soppe W.J.J., Viñegra de la Torre N., Albani M.C. (2021). The Diverse Roles of FLOWERING LOCUS C in Annual and Perennial Brassicaceae Species. Front. Plant Sci..

[39] Wu Z., Fang X., Zhu D., Dean C. (2020). Autonomous pathway: Flowering locus c repression through an antisense-mediated chromatin-silencing mechanism. Plant Physiol..

[40] Searle I., He Y., Turck F., Vincent C., Fornara F., Kröber S., Amasino R.A., Coupland G. (2006). The transcription factor FLC confers a flowering response to vernalization by repressing meristem competence and systemic signaling in *Arabidopsis*. Genes Dev..

[41] Fukazawa J., Ohashi Y., Takahashi R., Nakai K., Takahashi Y. (2021). Della degradation by gibberellin promotes flowering via gaf1-tpr-dependent repression of floral repressors in arabidopsis. Plant Cell.

[42] Balasubramanian S., Sureshkumar S., Lempe J., Weigel D. (2006). Potent induction of *Arabidopsis thaliana* flowering by elevated growth temperature. PLoS Genet..

[43] Reinhardt D., Mandel T., Kuhlemeier C. (2000). Auxin regulates the initiation and radial position of plant lateral organs. Plant Cell.

[44] Moubayidin L., Di Mambro R., Sabatini S. (2009). Cytokinin-auxin crosstalk. Trends Plant Sci..

[45] Li J., Li Y., Chen S., An L. (2010). Involvement of brassinosteroid signals in the floral-induction network of *Arabidopsis*. J. Exp. Bot..

[46] Jeyakumar J.M.J., Ali A., Wang W.M., Thiruvengadam M. (2020). Characterizing the role of the miR156-SPL network in plant development and stress response. Plants.

[47] Millar A.A., Lohe A., Wong G. (2019). Biology and function of miR159 in plants. Plants.

[48] Kim D.H., Sung S. (2017). Vernalization-Triggered Intragenic Chromatin Loop Formation by Long Noncoding RNAs. Dev. Cell.

[49] Zhang Y.Z., Yuan J., Zhang L., Chen C., Wang Y., Zhang G., Peng L., Xie S.S., Jiang J., Zhu J.K. (2020). Coupling of H3K27me3 recognition with transcriptional repression through the BAH-PHD-CPL2 complex in *Arabidopsis*. Nat. Commun..

[50] Luo X., Li X., Chen Z., Tian S., Liu Y., Shang Z., Chen L., Sun Y., Du J., He Y. (2025). A pair of readers of histone H3K4 methylation recruit Polycomb repressive complex 2 to regulate photoperiodic flowering. Nat. Commun..

[51] Kumar S.V., Wigge P.A. (2010). H2A.Z-Containing Nucleosomes Mediate the Thermosensory Response in *Arabidopsis*. Cell.

[52] Musialak-Lange M., Fiddeke K., Franke A., Kragler F., Abel C., Wahl V. (2025). The trehalose 6-phosphate pathway coordinates dynamic changes at the shoot apical meristem in *Arabidopsis thaliana*. Plant Physiol..

[53] Wang T., Sun M.-Y., Wang X.-S., Li W.-B., Li Y.-G. (2016). Over-expression of GmGIa-regulated soybean miR172a confers early flowering in transgenic *Arabidopsis thaliana*. Int. J. Mol. Sci..

[54] Simon R., Igeno M.I., Coupland G. (1996). Activation of floral meristem identity genes in *Arabidopsis*. Nature.

[55] Adrian J., Torti S., Turck F. (2009). From decision to commitment: The molecular memory of flowering. Mol. Plant.

[56] Bowman J.L., Moyroud E. (2024). Reflections on the ABC model of flower development. Plant Cell.

[57] Coen E.S., Meyerowitz E.M. (1991). The war of the whorls: Genetic interactions controlling flower development. Nature.

[58] Pelayo M.A., Yamaguchi N., Ito T. (2021). One factor, many systems: The floral homeotic protein AGAMOUS and its epigenetic regulatory mechanisms. Curr. Opin. Plant Biol..

[59] Zhao X., Li Y., Zhang M.M., He X., Ahmad S., Lan S., Liu Z.J. (2023). Research advances on the gene regulation of floral development and color in orchids. Gene.

[60] Lombardo F., Yoshida H. (2015). Interpreting lemma and palea homologies: A point of view from rice floral mutants. Front. Plant Sci..

[61] Hama E., Takumi S., Ogihara Y., Murai K. (2004). Pistillody is caused by alterations to the class-B MADS-box gene expression pattern in alloplasmic wheats. Planta.

[62] Lee Z., Kim S., Choi S.J., Joung E., Kwon M., Park H.J., Shim J.S. (2023). Regulation of Flowering Time by Environmental Factors in Plants. Plants.

[63] Sun F., Wang Y., Liu G., Fang D., Sun M., Bao Z., Ma F. (2024). Salt stress induces SFT expression to promote early flowering and inhibits floral organ development by disturbing cell cycle in tomato. Veg. Res..

[64] Barra L., Termolino P., Aiese Cigliano R., Cremona G., Paparo R., Lanzillo C., Consiglio M.F., Conicella C. (2021). Meiocyte Isolation by INTACT and Meiotic Transcriptome Analysis in Arabidopsis. Front. Plant Sci..

[65] Fartyal D., Crane A., Yasuor H. (2025). Pollen development under control and high-temperature stress: The role of plant hormones. Plant Stress.

[66] Zhang C., Wei L., Wang W., Qi W., Cao Z., Li H., Bao M., He Y. (2020). Identification, characterization and functional analysis of AGAMOUS subfamily genes associated with floral organs and seed development in Marigold (*Tagetes erecta*). BMC Plant Biol..

[67] Wang Y., Impa S.M., Sunkar R., Jagadish S.V.K. (2021). The neglected other half—Role of the pistil in plant heat stress responses. Plant Cell Environ..

[68] Hedhly A. (2011). Sensitivity of flowering plant gametophytes to temperature fluctuations. Environ. Exp. Bot..

[69] De Storme N., Geelen D. (2014). The impact of environmental stress on male reproductive development in plants: Biological processes and molecular mechanisms. Plant Cell Environ..

[70] Fernández V., Takahashi Y., Le Gourrierec J., Coupland G. (2016). Photoperiodic and thermosensory pathways interact through CONSTANS to promote flowering at high temperature under short days. Plant J..

[71] Cheung A.Y., Wu H.M. (2011). THESEUS 1, FERONIA and relatives: A family of cell wall-sensing receptor kinases?. Curr. Opin. Plant Biol..

[72] Palanivelu R., Tsukamoto T. (2012). Pathfinding in angiosperm reproduction: Pollen tube guidance by pistils ensures successful double fertilization. WIREs Dev. Biol..

[73] Nelson M.R., Band L.R., Dyson R.J., Lessinnes T., Wells D.M., Yang C., Everitt N.M., Jensen O.E., Wilson Z.A. (2012). A biomechanical model of anther opening reveals the roles of dehydration and secondary thickening. New Phytol..

[74] Kurusu T., Kuchitsu K., Tada Y. (2015). Plant signaling networks involving Ca^2+^ and Rboh/Nox-mediated ROS production under salinity stress. Front. Plant Sci..

[75] Sun T., Zhang Y. (2022). MAP kinase cascades in plant development and immune signaling. EMBO Rep..

[76] Shadel G.S., Horvath T.L. (2015). Mitochondrial ROS Signaling in Organismal Homeostasis. Cell.

[77] Iwata Y., Fedoroff N.V., Koizumi N. (2008). *Arabidopsis* bZIP60 is a proteolysis-activated transcription factor involved in the endoplasmic reticulum stress response. Plant Cell.

[78] Nawkar G.M., Lee E.S., Shelake R.M., Park J.H., Ryu S.W., Kang C.H., Lee S.Y. (2018). Activation of the transducers of unfolded protein response in plants. Front. Plant Sci..

[79] Rai G.K., Khanday D.M., Choudhary S.M., Kumar P., Kumari S., Martínez-Andújar C., Martínez-Melgarejo P.A., Rai P.K., Pérez-Alfocea F. (2024). Unlocking nature’s stress buster: Abscisic acid’s crucial role in defending plants against abiotic stress. Plant Stress.

[80] Okada K., Abe H., Arimura G.I. (2015). Jasmonates induce both defense responses and communication in monocotyledonous and dicotyledonous plants. Plant Cell Physiol..

[81] Tripathi S.K., Tuteja N. (2007). Integrated signaling in flower senescence: An overview. Plant Signal. Behav..

[82] Ariizumi T., Murase K., Sun T.P., Steber C.M. (2008). Proteolysis-Independent Downregulation of DELLA Repression in *Arabidopsis* by the Gibberellin Receptor GIBBERELLIN INSENSITIVE DWARF1. Plant Cell.

[83] Sawarkar R. (2022). Transcriptional lockdown during acute proteotoxic stress. Trends Biochem. Sci..

[84] Yang Y., Yu J., Qian Q., Shang L. (2022). Enhancement of Heat and Drought Stress Tolerance in Rice by Genetic Manipulation: A Systematic Review. Rice.

[85] Lohani N., Singh M.B., Bhalla P.L. (2022). Rapid Transcriptional Reprogramming Associated with Heat Stress-Induced Unfolded Protein Response in Developing *Brassica napus* Anthers. Front. Plant Sci..

[86] Pratx L., Wendering P., Kappel C., Nikoloski Z., Bäurle I. (2023). Histone retention preserves epigenetic marks during heat stress-induced transcriptional memory in plants. EMBO J..

[87] Begcy K., Dresselhaus T. (2018). Epigenetic responses to abiotic stresses during reproductive development in cereals. Plant Reprod..

[88] Forestan C., Farinati S., Zambelli F., Pavesi G., Rossi V., Varotto S. (2020). Epigenetic signatures of stress adaptation and flowering regulation in response to extended drought and recovery in *Zea mays*. Plant Cell Environ..

[89] Kakoulidou I., Avramidou E.V., Baránek M., Brunel-muguet S., Farrona S., Johannes F., Kaiserli E., Lieberman-lazarovich M., Martinelli F., Mladenov V. (2021). Epigenetics for crop improvement in times of global change. Biology.

[90] Ma Z., Lv J., Wu W., Fu D., Lü S., Ke Y., Yang P. (2023). Regulatory network of rice in response to heat stress and its potential application in breeding strategy. Mol. Breed..

[91] Hulsmans S., Rodriguez M., De Coninck B., Rolland F. (2016). The SnRK1 Energy Sensor in Plant Biotic Interactions. Trends Plant Sci..

[92] Bilichak A., Ilnytskyy Y., Woycicki R., Kepeshchuk N., Fogen D., Kovalchuk I. (2015). The elucidation of stress memory inheritance in *Brassica rapa* plants. Front. Plant Sci..

[93] Elkelish A., Alqudah A.M., Alsalamah S.A., Alqahtani H., Alhaithloul H.A.S., Fouda A., Ukozehasi C., Thabet S.G. (2025). Enhancing wheat resilience to combined drought and heat stress through genetic mapping of transgenerational stress memory. Chem. Biol. Technol. Agric..

[94] Virlouvet L., Avenson T.J., Du Q., Zhang C., Liu N., Fromm M., Avramova Z., Russo S.E. (2018). Dehydration Stress Memory: Gene Networks Linked to Physiological Responses During Repeated Stresses of *Zea mays*. Front. Plant Sci..

[95] Saharan B.S., Brar B., Duhan J.S., Kumar R., Marwaha S., Rajput V.D., Minkina T. (2022). Molecular and Physiological Mechanisms to Mitigate Abiotic Stress Conditions in Plants. Life.

[96] Nick P. (2024). Towards a grammar of plant stress: Modular signalling conveys meaning. Theor. Exp. Plant Physiol..

[97] Lakhneko O., Stasik O., Škultéty Ľ., Kiriziy D., Sokolovska-Sergiienko O., Kovalenko M., Danchenko M. (2023). Transient drought during flowering modifies the grain proteome of bread winter wheat. Front. Plant Sci..

[98] Lawas L.M.F., Shi W., Yoshimoto M., Hasegawa T., Hincha D.K., Zuther E., Jagadish S.V.K. (2018). Combined drought and heat stress impact during flowering and grain filling in contrasting rice cultivars grown under field conditions. Field Crops Res..

[99] Talukder A., McDonald G.K., Gill G.S. (2014). Effect of short-term heat stress prior to flowering and early grain set on the grain yield of wheat. Field Crops Res..

[100] Prasad P.V.V., Djanaguiraman M., Perumal R., Ciampitti I.A. (2015). Impact of high temperature stress on floret fertility and individual grain weight of grain sorghum: Sensitive stages and thresholds for temperature and duration. Front. Plant Sci..

[101] Caspersen L., Schiffers K., Picornell A., Egea J.A., Delgado A., El Yaacoubi A., Benmoussa H., Rodrigo J., Fadón E., Mimoun M.B. (2025). Contrasting responses to climate change—Predicting bloom of major temperate fruit tree species in the Mediterranean region and Central Europe. Agric. For. Meteorol..

[102] Barman M., Tenhaken R., Dotterl S. (2024). Negative and sex-specific effects of drought on flower production, resources and pollinator visitation, but not on floral scent in monoecious *Cucurbita pepo*. New Phytol..

[103] Kazan K., Lyons R. (2016). The link between flowering time and stress tolerance. J. Exp. Bot..

[104] Abbas A.M., Alomran M.M., Alharbi N.K., Novak S.J. (2023). Suppression of Seedling Survival and Recruitment of the Invasive Tree *Prosopis juliflora* in Saudi Arabia through Its Own Leaf Litter: Greenhouse and Field Assessments. Plants.

[105] Yang L., Fang S., Liu L., Zhao L., Chen W., Li X., Xu Z., Chen S., Wang H., Yu D. (2025). WRKY transcription factors: Hubs for regulating plant growth and stress responses. J. Integr. Plant Biol..

[106] Kiyono H., Katano K., Suzuki N. (2021). Links between Regulatory Systems of ROS and Carbohydrates in Reproductive Development. Plants.

[107] Liu H., Zeng B., Zhao J., Yan S., Wan J., Cao Z. (2023). Genetic Research Progress: Heat Tolerance in Rice. Int. J. Mol. Sci..

[108] Mei F., Chen B., Du L., Li S., Zhu D., Chen N., Zhang Y., Li F., Wang Z., Cheng X. (2022). A gain-of-function allele of a DREB transcription factor gene ameliorates drought tolerance in wheat. Plant Cell.

[109] Husaini A.M. (2022). High-value pleiotropic genes for developing multiple stress-tolerant biofortified crops for 21st-century challenges. Heredity.

[110] Yamaguchi N., Ito T. (2021). JMJ Histone Demethylases Balance H3K27me3 and H3K4me3 Levels at the HSP21 Locus during Heat Acclimation in *Arabidopsis*. Biomolecules.

[111] Borghi M., Fernie A.R. (2017). Floral metabolism of sugars and amino acids: Implications for pollinators’ preferences and seed and fruit set. Plant Physiol..

[112] Hayat S., Hayat Q., Alyemeni M.N., Wani A.S., Pichtel J., Ahmad A. (2012). Role of proline under changing environments: A review. Plant Signal. Behav..

[113] Mo W., Zheng X., Shi Q., Zhao X., Chen X., Yang Z., Zuo Z. (2024). Unveiling the crucial roles of abscisic acid in plant physiology: Implications for enhancing stress tolerance and productivity. Front. Plant Sci..

[114] Cai H., Liu K., Ma S., Su H., Yang J., Sun L., Liu Z., Qin Y. (2025). Gibberellin and cytokinin signaling antagonistically control female-germline cell specification in *Arabidopsis*. Dev. Cell.

[115] Twell D., Yamaguchi J., McCormick S. (1990). Pollen-specific gene expression in transgenic plants: Coordinate regulation of two different tomato gene promoters during microsporogenesis. Development.

[116] Steffen J.G., Kang I.H., Macfarlane J., Drews G.N. (2007). Identification of genes expressed in the *Arabidopsis* female gametophyte. Plant J..

[117] Ye C., Tenorio F.A., Redoña E.D., Morales–Cortezano P.S., Cabrega G.A., Jagadish K.S.V., Gregorio G.B. (2015). Fine-mapping and validating qHTSF4.1 to increase spikelet fertility under heat stress at flowering in rice. Theor. Appl. Genet..

[118] Rani A., Devi P., Jha U.C., Sharma K.D., Siddique K.H.M., Nayyar H. (2020). Developing Climate-Resilient Chickpea Involving Physiological and Molecular Approaches with a Focus on Temperature and Drought Stresses. Front. Plant Sci..

[119] Li R., Liu C., Zhao R., Wang L., Chen L., Yu W., Zhang S., Sheng J., Shen L. (2019). CRISPR/Cas9-Mediated SlNPR1 mutagenesis reduces tomato plant drought tolerance. BMC Plant Biol..

[120] Yu X., Liu Z., Sun X. (2023). Single-cell and spatial multi-omics in the plant sciences: Technical advances, applications, and perspectives. Plant Commun..

[121] Lee T.A., Illouz-Eliaz N., Nobori T., Xu J., Jow B., Nery J.R., Ecker J.R. (2025). A single-cell, spatial transcriptomic atlas of the *Arabidopsis* life cycle. Nat. Plants.

[122] Cembrowska-Lech D., Krzemińska A., Miller T., Nowakowska A., Adamski C., Radaczyńska M., Mikiciuk G., Mikiciuk M. (2023). An Integrated Multi-Omics and Artificial Intelligence Framework for Advance Plant Phenotyping in Horticulture. Biology.

